# Neuroimaging in Hereditary Spastic Paraplegias: Current Use and Future Perspectives

**DOI:** 10.3389/fneur.2018.01117

**Published:** 2019-01-16

**Authors:** Felipe Franco da Graça, Thiago Junqueira Ribeiro de Rezende, Luiz Felipe Rocha Vasconcellos, José Luiz Pedroso, Orlando Graziani P. Barsottini, Marcondes C. França

**Affiliations:** ^1^Department of Neurology and Neuroimaging Laboratory, University of Campinas (UNICAMP), Campinas, Brazil; ^2^Institute of Neurology, Federal University of Rio de Janeiro (UFRJ), Rio de Janeiro, Brazil; ^3^Department of Neurology, Federal University of São Paulo (UNIFESP), São Paulo, Brazil

**Keywords:** MRI, hereditary spastic paraplegia, diagnosis, DTI, spinal cord

## Abstract

Hereditary spastic paraplegias (HSP) are a large group of genetic diseases characterized by progressive degeneration of the long tracts of the spinal cord, namely the corticospinal tracts and dorsal columns. Genotypic and phenotypic heterogeneity is a hallmark of this group of diseases, which makes proper diagnosis and management often challenging. In this scenario, magnetic resonance imaging (MRI) emerges as a valuable tool to assist in the exclusion of mimicking disorders and in the detailed phenotypic characterization. Some neuroradiological signs have been reported in specific subtypes of HSP and are therefore helpful to guide genetic testing/interpretation. In addition, advanced MRI techniques enable detection of subtle structural abnormalities not visible on routine scans in the spinal cord and brain of subjects with HSP. In particular, quantitative spinal cord morphometry and diffusion tensor imaging look promising tools to uncover the pathophysiology and to track progression of these diseases. In the current review article, we discuss the current use and future perspectives of MRI in the context of HSP.

## Introduction

Hereditary spastic paraplegias (HSP) are a large group of genetic diseases characterized by progressive degeneration of the long tracts of the spinal cord, namely the corticospinal tracts and dorsal columns. Patients typically present with lower limb-predominant spasticity and weakness leading to gait abnormalities. Sensory deficits and urinary complaints are also frequently found (1). In the realm of neurogenetics, HSP is perhaps the condition with the most striking genetic heterogeneity. It may segregate as an autosomal dominant, autosomal recessive or X-linked trait. There are now around 70 loci and 60 genes associated to different forms of HSP (2). For the practicing clinician, this represents a diagnostic challenge and poses difficulty for proper therapeutic management as well as genetic counseling.

Neuroimaging is a powerful tool that enables the structural and functional assessment of the central nervous system (CNS). In particular, advanced techniques of magnetic resonance imaging (MRI) are able to provide detailed microstructural and biochemical information of the CNS; they have proven useful to uncover abnormalities in closely related heredodegenerative diseases, such as spinocerebellar ataxias and amyotrophic lateral sclerosis (3, 4). In the context of HSPs, MRI may assist in the phenotypic characterization and therefore help in the genetic testing approach (5). More recently, some studies using advanced and quantitative techniques have shown microstructural white matter abnormalities in HSP, not detectable with routine MRI sequences (6, 7). These results brought novel insights into the pathophysiology of HSPs and raised the possibility of using MRI as a biomarker to track disease progression. In this review article, we will discuss the available data concerning neuroimaging in HSP.

## Routine MRI in HSP: Diagnostic Clues for Specific HSP Subtypes

The precise diagnosis of HSPs, considering the great genetic variability with often similar phenotypes, is challenging. However, as with any neurological disease, a combination of detailed history, assessment of inheritance pattern, accompanying symptoms and physical examination is paramount to reduce the number of hypotheses and to optimize genetic testing (8).

In patients with spastic paraparesis, MRI is essential, initially to rule out usual causes of paraplegia such as compressive, inflammatory, infectious, or vascular myelopathies. Once the diagnosis of HSP is the most likely, neuroimaging may help in establishing the subtype.

**Figure 1:**
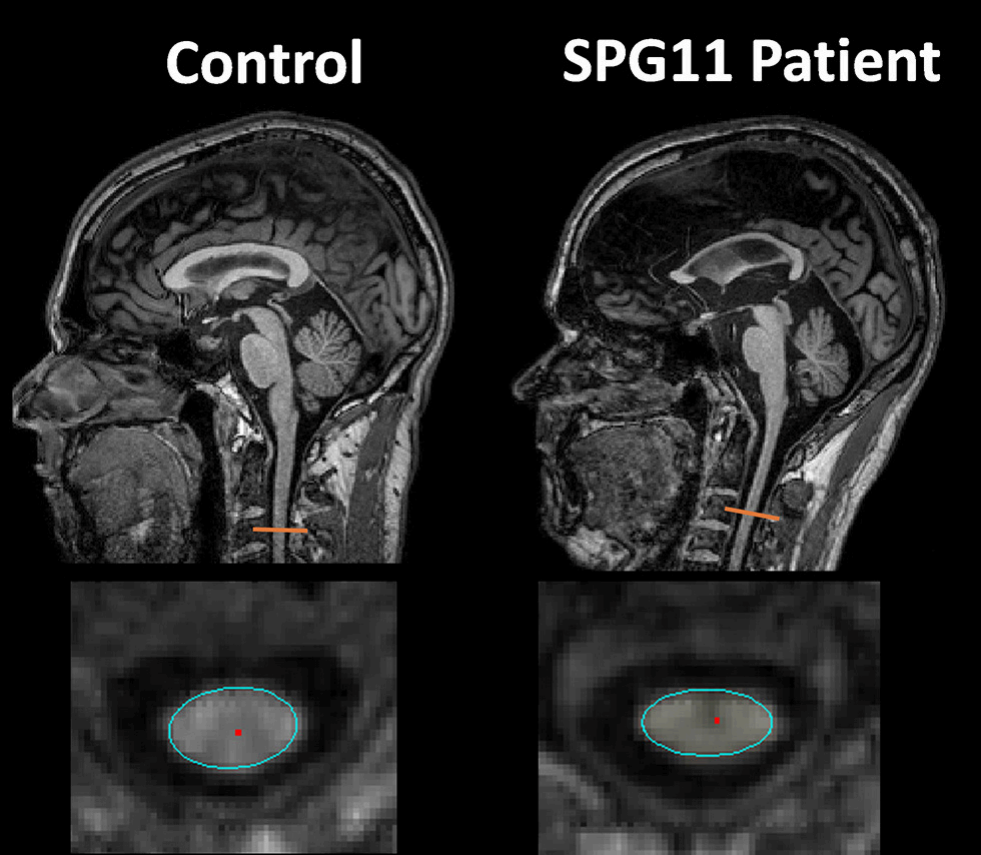
Sagittal and axial T1 weighted images showing cervical spinal cord atrophy and antero-posterior flattening in a patient with SPG11 (**right** column) compared to a healthy control (**left** column).

The evaluation of spinal cord images, although very useful to rule out differential diagnoses, will rarely provide relevant tips for establishing the HSP subtype. Considering the pathophysiology of this group of diseases (involving axonal degeneration of long motor tracts), it is easy to understand why spinal cord volumetric reduction is found in most patients—Figure 1 (9, 10). Studies looking at the cervical and thoracic spinal cord cross-sectional areas in patients with ADP HSP highlighted significant alterations in all subtypes evaluated (SPG3A, 4, 6, and 8), particularly for SPG 6 and SPG 8 (without direct correlation between the degree of atrophy and clinical deficits) (9). Nevertheless, studies with SPG11, an AR subtype of HSP, demonstrated a direct correlation between disease duration and the severity of spinal cord volumetric reduction (6). Such atrophy, however, is not always evident in visual analyses and even if it is, this characteristic is not exclusive for HSPs and may also be found, for example, in acquired motor neuron diseases (4). Anecdotal reports of spinal cord abnormalities in HSPs have been also described. For example, prominent atrophy of the gracile fasciculus leading to visible enlargement of the ependymal canal in patients with AR HSP was reported (without specifying the subtype) (10). A Single patient with SPG 56 and hydromyelia revealed in MRI scans was described, suggesting that this finding was part of the general clinical picture and not the cause of paraparesis itself (11).

**Figure 2:**
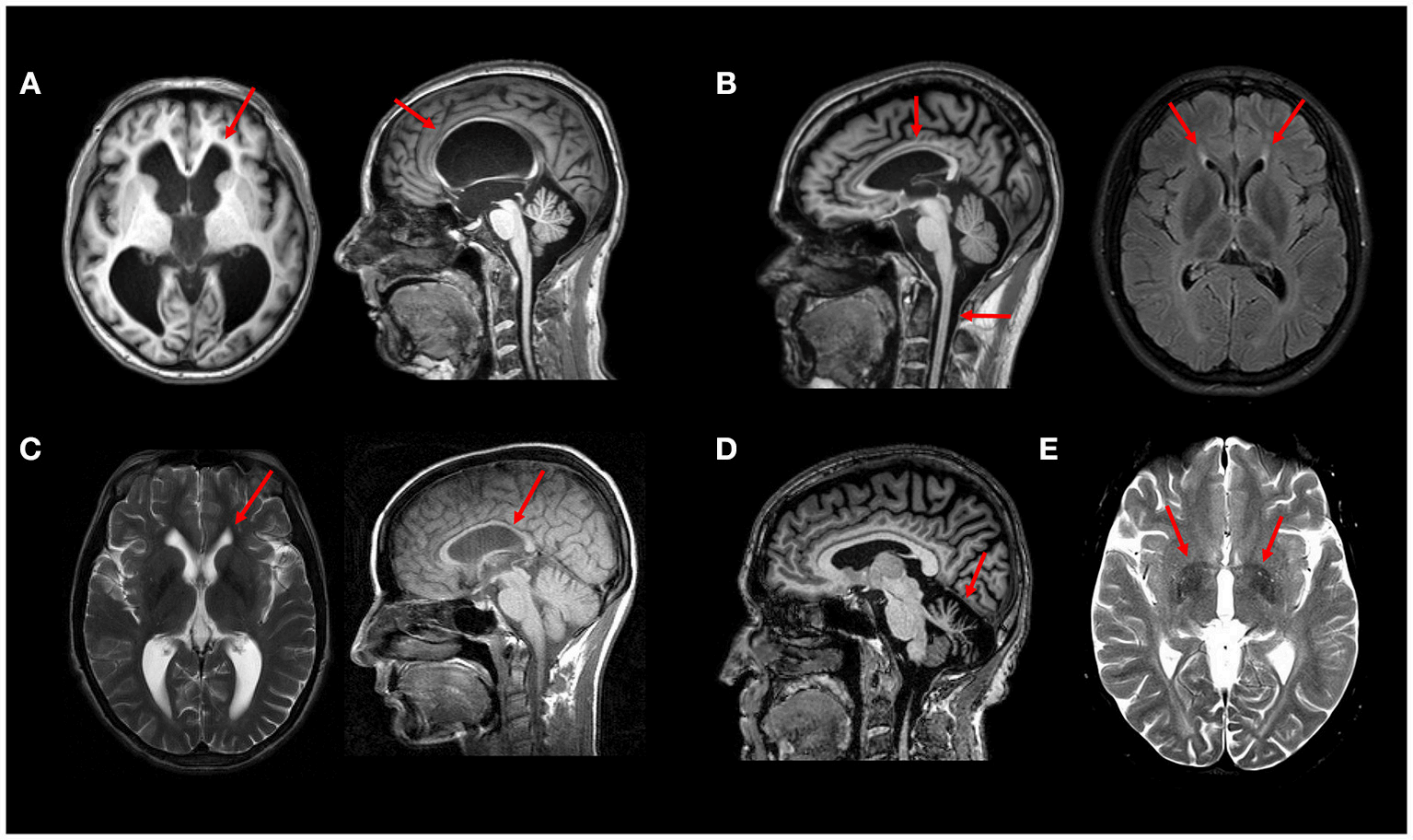
Typical MRI findings in distinct HSP subtypes—**(A)** Axial and sagittal T1W images showing hydrocephalus due to aqueductal stenosis in a patient with SPG4. This finding is rather unusual in SPG4, but relatively frequent in SPG1 patients. **(B)** Sagittal T1W image showing thin corpus callosum and spinal cord atrophy in a patient with SPG11; Axial FLAIR image showing the “ear-of-the-lynx” sign in the same patient. **(C)** Thin corpus callosum (T1W image) and subtle “ear-of-the-lynx” sign (T2W image) in a patient with SPG15. **(D)** Sagittal T1W image revealing marked cerebellar atrophy in a patient with SPG7. **(E)** White matter hyperintensities and T2 hypointense signal at the globi pallidi in a 21-years old patient with SPG35 (T2W image). All images were obtained during routine clinical care of these patients at UNICAMP and UNIFESP hospitals.

Unlike spinal cord imaging, brain MRI may show peculiar findings that may provide relevant clues for a specific genetic subtype. The presence of a thin corpus callosum (TCC) in a complicated AR HSP phenotype has been used to characterize a certain group of patients (HSP-TCC) (12). It was first considered that this MRI pattern was specific for SPG11 (Figure 2B), but it is now clear that other genotypes may share the same imaging profile. A comprehensive genetic evaluation of an Italian cohort of patients with HSP-TCC (*n* = 61) was performed and found SPG11 to be the most frequent subtype (26.2%), followed by SPG15 (14.8%), SPG35 (5%), and SPG48 (3%) (13). Other less frequent causes of HSP-TCC are SPG 4, 7, 18, 21, 46, 47, 49, and 54 (8). A new mutation for the SPG3A gene also presented with TCC (14).

SPG11 itself has the most classic neuroimaging finding in this group of diseases (15). The so-called “ear-of-the-lynx sign” (Figure 2B) is characterized by an abnormality at the forceps minor of the corpus callosum (genum fibers), which appears hyperintense on T2-FLAIR-weighted and hypointense on T1-weighted images (16). This radiological sign may also be present in another form of complicated AR HSP: SPG 15 (Figure 2C). The visual evaluation of T2-FLAIR-weighted images has sensitivity and specificity for these 2 HSP subtypes as high as 94 and 97%, respectively (Pascual et al. Accepted). In a compatible clinical setting, this should raise a strong suspicion for the above described subtypes, even in the early stages of the disease.

SPG1, also known as MASA syndrome (Mental retardation, Aphasia, Shuffling gait, and Adducted thumbs) is a disease allelic to X-linked aqueductal stenosis or hydrocephalus and may also present with enlarged ventricles. Therefore, in a patient with X-linked inheritance and hydrocephalus, SPG1 should be the main hypothesis (17). Hydrocephalus has been also rarely described in SPG4 patients (as did subarachnoid cysts) Figure 2A (18, 19).

SPG2, on the other hand, is also X-linked and shares phenotypic similarities with an allelic disease, Pelizaeus Merzbacher disease (PMD). Although leukoencephalopathy is a much more evident feature in PMD, it may be also present in SPG2 (20). Multifocal areas of T2 white matter hyperintensities are also a possible presenting feature (sometimes they may even resemble those seen in multiple sclerosis) (21). This last MRI pattern has been reported in some AR-HSP subtypes, such as SPG 5, 21, and 35 (22–24). Other forms of autosomal recessive HSP with white matter disease include SPG11, SPG22, SPG26, SPG44, SPG45, SPG47, SPG50, SPG51, SPG52, SPG54, SPG56, SPG63, SPG64, and SPG 67 (25).

Considering the frequent association of extrapyramidal symptoms in patients with complicated HSPs, changes in the basal ganglia have been conspicuously described, mainly as volumetric abnormalities. Visually perceptible changes, although rare, exist and may assist in the diagnostic definition. In particular, one should remember that some HSP subtypes have basal ganglia iron deposition revealed as hypointense signal lesions in T2, T2^*^ or Susceptibility weighted images. Patients with SPG28, SPG35, and SPG43 (24–27) may present NBIA phenotype with T2 hypointense signal at the globus pallidus (Figure 2E).

It is worth remembering, however, that other NBIAs also manifest with spasticity and similar imaging findings, and should be considered in the differential diagnosis. In addition to structural changes in the basal ganglia (BG), reduced dopaminergic binding with TRODAT has been described in SPG11 and SPG7 patients with complicated HSP phenotype and dopa-responsive parkinsonism (28, 29). Besides BG abnormalities, SPG7 may also present marked and diffuse cerebellar atrophy—Figure 2D (30).

**Figure 3:**
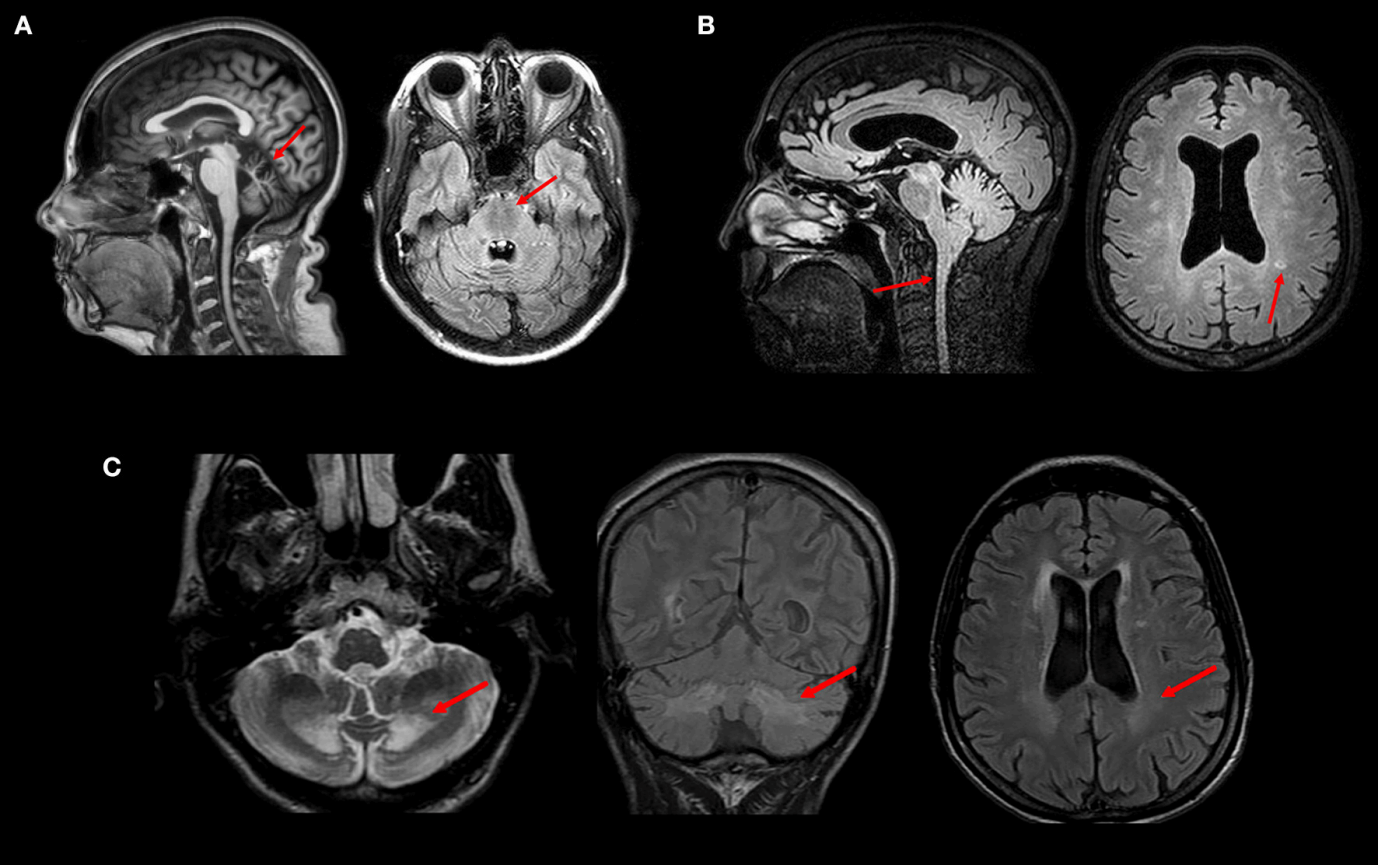
MRI and clinical findings in patients with inherited diseases that may mimic HSP—**(A)** Superior vermis atrophy (T1W image) and linear hypointensities (FLAIR image) in the pons in a patient with ARSACS. **(B)** Spinal cord atrophy and white matter hyperintensities (FLAIR images) in a patient with Adrenomyeloneuropathy. **(C)** Peridentate and periventricular white matter signal changes (FLAIR and T2W images) in a patient with Cerebrotendinous Xanthomatosis. All images were obtained during routine clinical care of these patients at UNICAMP and UNIFESP hospitals.

Finally, neuroimaging may assist in the differential diagnosis of other diseases that are not included in the HSP group, but which may manifest with a predominance of spastic symptoms. The presence of a marked cerebellar atrophy with AD history may raise the suspicion for spinocerebellar ataxias (SCA), and some may manifest with predominantly paraparetic forms such as SCA3 (Machado-Joseph Disease) and SCA 7 (31). If cerebellar atrophy is associated with superior vermis atrophy and linear hypointensities in the pons, ARSACS is a possibility—Figure 3A (32). Cerebrotendinous Xantomatosis in adults may also display paraparetic symptoms and MRI abnormalities reveal diffuse atrophy, white matter signal changes and focal peri-dentate cerebellar lesions (Figure 3C); increased lactate with decreased n-acetylaspartate are also seen in MR spectroscopy (33, 34). X-Linked adrenomyeloneuropathy is also a differential diagnosis even in women (about 20% of female carriers develop spastic paraparesis in middle age); typical findings are predominantly posterior white matter T2 hyperintensities and reduced spinal cord volume are key features—Figure 3B (35). Adult-onset Langerhans cell histiocytosis (LCH) is a rare proliferative disorder with neurologic symptoms, including cerebellar ataxia and spastic paraparesis (36). LCH brain MRI can demonstrate globus pallidus/dentate nucleus T1 hyperintensity as well as brainstem and cerebellum T2 hyperintensity.

**Figure 4:**
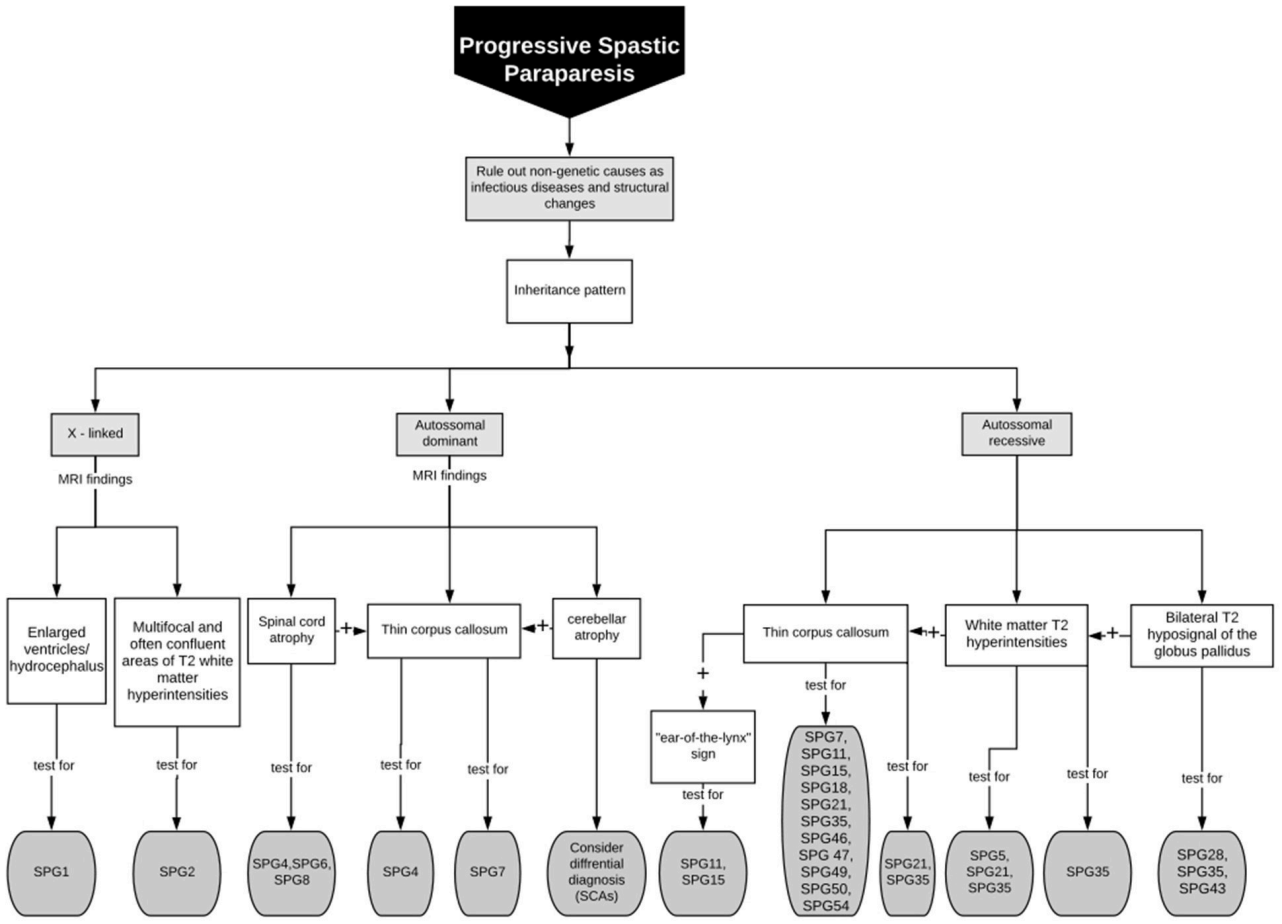
Flowchart to guide genetic testing for HSP based on MRI findings.

Despite the above discussed neuroimaging clues for HSP diagnosis, (and the several new descriptions being published in the area) (37), these findings are rarely sensitive or specific. So that their absence should not exclude a diagnostic hypothesis of HSP raised by clinical findings. One must always proceed to diagnostic genetic testing in cases of high clinical suspicion. Different neuroimaging features and their related genetic HSP subtypes herein described are summarized in Table 1, Figure 4 is a flowchart to guide genetic testing for HSP based on MRI findings.

**Table 1:** Neuroimaging findings in different HSP subtypes.

**Characteristics in neuroimaging**	**HSP subtypes**
Thin corpus callosum	SPG4, SPG7, SPG11, SPG15, SPG18, SPG21, SPG35, SPG46, SPG 47, SPG49, SPG50, SPG54
Prominent spinal cord atrophy	SPG4, SPG6, SPG8
Ear-of-the-lynx sign	SPG11, SPG15
Enlarged ventricles/hydrocephalus	SPG1, SPG4 (rarely)
White matter T2 hyperintensities	SPG2, SPG11, SPG5, SPG35
Bilateral T2 hyposignal of the globus pallidus	SPG28, SPG35, SPG43
Thoracic spinal cord hydromelia	SPG56

## Advanced MRI in HSP: Understanding Pathophysiology and Potential Role as a Biomarker

The widely variable clinical presentation of patients with HSP suggests that damage is not restricted to the corticospinal tracts in most subtypes of the disease. In this scenario, studies with advanced MRI have been used to investigate the real extension of cerebral damage in the disease (6, 7, 38–47). Such techniques are much more sensitive to capture subtle anatomical abnormalities than pure visual analyses. Moreover, they enable assessment of the CNS in three-dimensions, quantitatively and non-invasively. There are not many image-based studies in HSP and most of them relied upon small cohorts. These reports looked mostly at cerebral structural abnormalities, but some of them also investigated spinal cord morphometry (9, 42, 43). Very few assessed functional abnormalities in HSP (38, 46, 47).

### Cerebral Structural Abnormalities in HSP

Gray matter abnormalities in HSP—including the cortex and basal ganglia—were assessed using different techniques, such as voxel-based morphometry (VBM, *n* = 3), cortical thickness analyses (FreeSurfer, *n* = 1) and atlas-based segmentation volumetry (*n* = 1). The white matter assessment was performed either through volumetric analysis or through diffusion tensor imaging. To assess the WM volume, two studies used the VBM approach. In contrast, the diffusion images ware assessed using tractography (*n* = 2) and tract-based spatial statistics (*n* = 3). Only 5 studies evaluated simultaneously cerebral GM and WM in the same cohort of patients (6, 7, 40, 42, 43).

**Figure 5:**
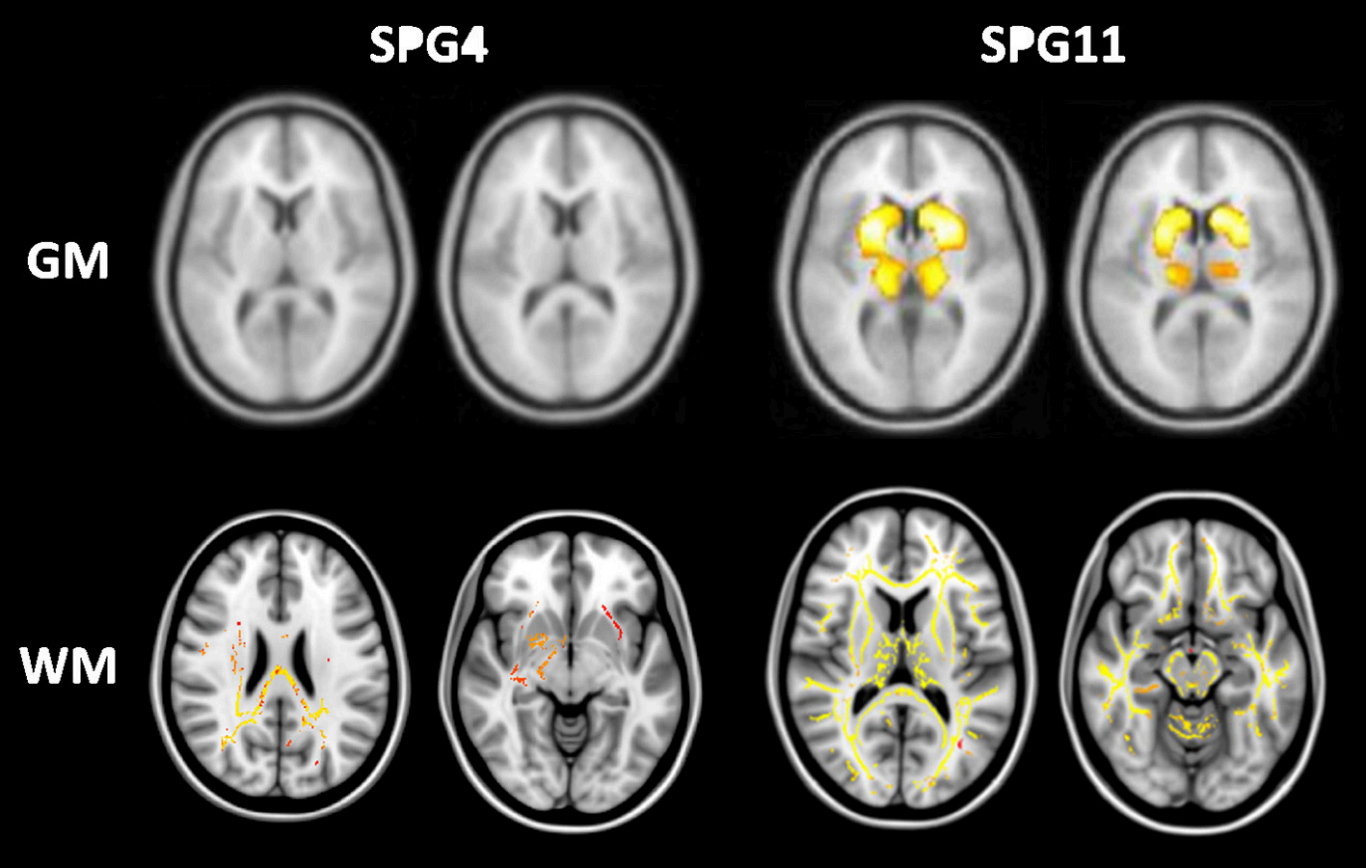
Structural signature for pure (SPG4) and complicated (SPG11) subtypes of HSP stratified for cerebral gray (GM-upper lane) and white matter (WM-lower lane). Regions highlighted in red-yellow are those found to be abnormal in each subtype of the disease. GM and WM results were obtained after comparison with matched healthy controls using voxel-based morphometry and tract-based spatial statistics, respectively. Adapted from references (40) and (42) with permission of the journals.

Advanced neuroimaging techniques are also useful to identify the distinct patterns of damage across the whole phenotypic and genotypic spectrum of HSPs. This might give us relevant insights into the genotype-phenotype correlations and ultimately into the pathophysiology of these disorders. Most studies split HSPs into pure and complicated forms in an attempt to identify different structural signatures for these two subgroups. The former group is characterized by widespread WM abnormalities, but essentially preserved cerebral GM (7, 37, 42). In contrast, the complicated forms, beyond WM damage, show GM volumetric reduction in the basal ganglia and cerebral cortex, particularly at the precentral, paracentral, cingulate, and parahippocampal gyri (6, 43). Overall, these imaging findings are in line with the few pathological reports available (48, 49). At this point, basal ganglia damage appears to be specific for complicated HSP and helps to explain some phenotypic features found in this subgroup, such as parkinsonism and dystonia (Figure 5). Cognitive decline, often severe, is frequent in complicated HSPs and is also related to cerebral GM atrophy. The absence of GM damage in pure HSP also explains why cognitive functions tend to be preserved in this subtype of the disease. Some patients with SPG4—a pure subtype of HSP—develop major cognitive deficits (50). The neuroanatomical substrate for such deficits still deserve investigation, but may be related to subcortical WM involvement, which is prominent in SPG4 (Figure 5).

There are very few imaging studies looking at structural abnormalities in specific genetic subtypes of HSP. As mentioned previously, most authors combined heterogeneous series of patients with different molecular diagnoses and then divided them into pure vs. complicated forms. This was mostly due to the rarity of HSPs in general. More recently, a couple of papers came out with reasonably large cohort sizes of SPG4 and especially SPG11 (6, 7, 38, 42, 51). In SPG4, results largely overlap those reported for pure HSP in general—widespread WM damage (diffusion and volumetric damage) with mild or absent cerebral GM atrophy (7, 42, 51). Interestingly, the mutational profile at *SPAST* seems to play a role in neuroradiological findings. Indeed, Rezende et al. (42) identified that SPG4 patients with missense mutations had more severe corticospinal tract diffusivity abnormalities than patients with non-sense/frameshift variants (42). This finding is quite interesting because both subgroups were matched for age and disease duration. Distinct neurophysiologic results (somatosensory evoked potentials) were also found in patients with missense and nonsense *SPAST* mutations (52). In a recent publication of MRI findings and clinical correlates in a multicentric Brazilian cohort of 25 patients with SPG11, authors found widespread cerebral WM (diffusion and volumetric damage) and deep GM abnormalities in these subjects (6). Basal ganglia atrophy correlated with cognitive deficits and also motor function in these subjects. Surprisingly, no clinical correlate for WM damage was found. Furthermore, GM, but not WM, damage was significantly correlated with disease duration. Overall, these results indicate that *KIAA1840* mutations impact cerebral GM and WM in distinct ways.

### Cerebral Functional Imaging in HSP

Functional imaging offers the opportunity to investigate how cerebral physiology changes in patients with HSP. To tackle this question, functional MRI (fMRI) and nuclear medicine techniques, such as PET or SPECT, have been used (43, 44, 49–60). The first one relies upon dynamic measurement of the BOLD signal using specific MRI acquisition sequences, whereas the others depend on the detection of radiation emitted by labeled radioligands.

There are 3 studies using fMRI in subjects with HSP (39, 46, 47). In 2 of them, authors employed a motor task to investigate how different cerebral regions are activated in the disease (39, 47). Both studies found abnormal primary motor cortex activation in patients with HSP in comparison to healthy controls; surprisingly, one study found increased whereas the other found reduced activation. In a resting state fMRI study, Liao et al recently reported abnormal functional connectivity between middle frontal and orbitofrontal gyri in patients with SPG4 (49). These authors also found that the amplitude of low frequency fluctuations of the BOLD signal at the right precentral gyrus was increased in the disease. This last parameter was significantly correlated with disease severity.

**Figure 6:**
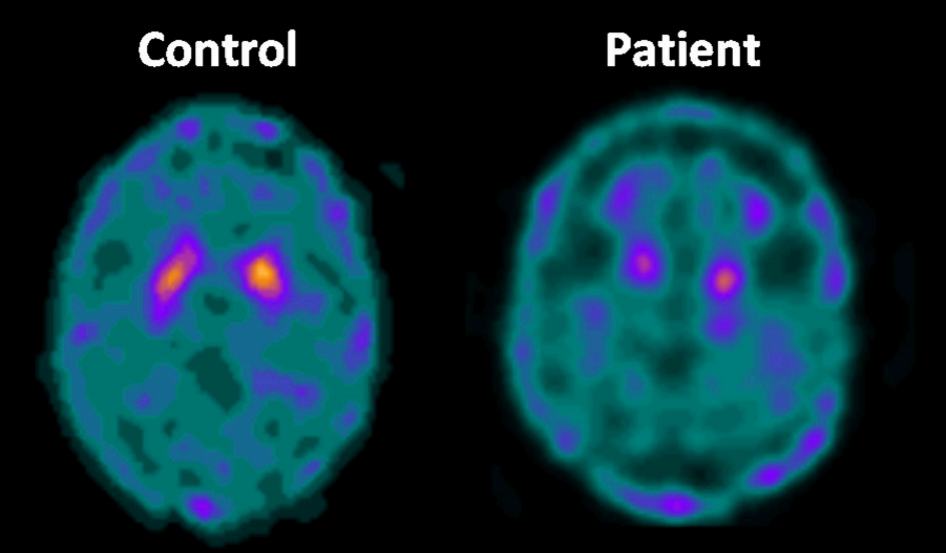
Axial DAT-scan with ^99m^Tc-TRODAT showing reduced striatal uptake in a patient with SPG11 in comparison to a healthy control.

Cerebral metabolism in HSP was assessed in a few studies using PET and the [18F]-Fluorodeoxyglucose tracer—Table 2 (53–63). Cortical hypometabolism was the usual finding, but the regions affected were rather heterogeneous across the different reports. Such discrepancy is possibly due to the small sample sizes (most studies were single case reports or small series). Parkinsonism and dystonia are now recognized as part of the phenotypic spectrum of some HSP subtypes (29). Hence, some authors employed dopamine transporter (DAT) radiotracers to evaluate the nigrostriatal pathway in the disease using either PET or SPECT (Table 2). In a recent publication, Faber et al. found reduced bilateral striatal DAT uptake to be a universal phenomenon in a cohort of 22 patients with SPG11 (Figure 6). Even patients without parkinsonism had clearly abnormal results (63).

**Table 2:** Studies using nuclear medicine techniques in different HSP subtypes.

**Author (year)**	**SPG**	***N (pts/ctls)***	**Technique**	**Tracer**	**Major findings**	**References**
Nielsen et al., 2004	SPG4	1/6	PET	${\text{H}}_{2}^{15}$O	↓Regional cerebral perfusion at the cerebellar hemispheres and left fusiform gyrus	(53)
Orlacchio et al., 2005	SPG4	6/–	SPECT	[^99m^Tc]ECD	↓Frontal/Frontoparietal perfusion	(54)
Scheuer et al., 2005	SPG4	18/18	PET	${\text{H}}_{2}^{15}$O	↓Regional cerebral perfusion at left fronto-temporal cortex	(55)
Hehr et al., 2007	SPG11	3/–	PET	[^18^F]FDG	↓ Metabolism at frontoparietal cortices and thalami (progressive)	(56)
Samaranch et al., 2008	SPG11	3/–	PET	[^18^F]FDG	↓ Metabolism at paracentral cortices and thalami	(57)
Órlen et al., 2008	SPG11	2/–	PET	[^18^F]FDG	↓ Metabolism at sensorimotor cortices and thalami. Absent uptake at the anterior cingulum and corpus callosum	(58)
Criscuolo et al., 2009	SPG5	3/–	PET	[^18^F]FDG	↓ Metabolism at the cerebellar vermis in one of the patients	(59)
Anheim et al., 2009	SPG11	2/–	SPECT	[^123^I]Ioflupane	↓ Bilateral striatal uptake of the tracer	(29)
Goizet et al., 2009	SPG15	1/–	SPECT	[^99m^Tc]ECD	↓ Frontotemporal perfusion	(60)
Svenstrup et al., 2010	SPG2	2/–	PET	[^18^F]FDG	Normal findings in both patients	(20)
Terada et al., 2013	SPG3A	2/–	PET	[^18^F]FDG	↓ Metabolism in dorsolateral and medial frontal cortices	(61)
Ma et al., 2014	SPG11	1/-	PET	[^18^F]FDG	↓ Metabolism in both cerebellar hemispheres	(62)
Pedroso et al., 2018	SPG7	1/–	SPECT	[^99m^Tc]TRODAT-1	↓ Bilateral caudate and putaminal uptake of the tracer	(28)
Faber et al., 2018	SPG11	22/19	SPECT	[^99m^Tc]TRODAT-1	↓ Bilateral caudate and putaminal uptake of the tracer. Uptake correlated with motor and cognitive scores	(63)

*PET, Positron emission tomography; SPECT, Single photon emission computerized tomography*.

Magnetic resonance spectroscopy is another imaging technique that enables the biochemical characterization of disease-related cerebral abnormalities. The most frequently reported abnormality in patients with HSP is reduction of N-acetylaspartate (a marker for neuronal/axonal function and viability) in the subcortical WM, but other findings have been also described—Table 3 (64–69).

**Table 3:** Studies using proton magnetic resonance spectroscopy (1H-MRS) in different HSP subtypes.

**Author (year)**	**SPG**	***N (pts/ctls)***	**Scanner**	**Major findings**	**References**
Pizzini et al., 2003	SPG2	1/3	1.5T	↓NAA/Cr ratio in the deep subcortical white matter	(64)
Erichsen et al., 2009	SPG4	8/8	1.5T	↓Cho/Cr ratio in the primary motor cortices	(65)
Svenstrup et al., 2010	SPG2	2/-	1.5T	↑mI/Cr ratio in the parieto-occipital white matter and centrum semiovalle	(20)
Stromillo et al., 2011	SPG11	10/10	1.5T	↓NAA/Cr ratio in the deep subcortical white matter	(38)
Schuurs-Hoeijmakers et al., 2012	SPG54	5/-	1.5T	Lipid peak at 1.3ppm in the thalami and basal ganglia	(66)
Roos et al., 2014	SPG5	2/-	3T	↑mI/Cr ratio in the parieto-occipital white matter	(67)
Fraidakis et al., 2016	SPG11	1/-	3T	↓NAA/Cr ratio in the deep subcortical white matter	(68)
Schneider-Gold et al., 2017	SPG11	2/2	3T	↓NAA/Cr and NAA/mI ratios in the corpus callosum. Normal cerebellar spectra.	(69)

*NAA, N-acetylaspartate; Cr, Creatine; mI, Myoinositol; Cho, Choline*.

### Quantitative Spinal Cord Imaging in HSP

The spinal cord is perhaps the major target of degeneration in HSPs and MRI studies published so far found it to be atrophic but without antero-posterior flattening in pure and complicated forms of the disease (6, 9, 42, 43, 70). Most reports assessed cervical spinal cord cross sectional area, but at least one also reported reduced thoracic dimensions (70). Postmortem studies corroborate these neuroimaging findings (48, 49). Faber et al. (6) recently found in a large SPG11-cohort that spinal cord area had an inverse correlation with disease duration (6). This argues in favor of a degenerative process taking place. However, it is possible that some sort of developmental issue also occurs. In a closely related heredodegenerative disorder—Friedreich's ataxia-imaging and pathological data support the later hypothesis (71, 72). Further studies in young patients with HSP should be done to check whether the same holds true in this group of diseases.

### Potential Neuroimaging Biomarkers

As basic research progresses and potential disease-modifying treatments become available, biomarkers will be needed to assist in the design of clinical trials for HSPs. In this context, some MRI-derived metrics look promising because they presented significant correlation with clinical data (disease duration and severity). In terms of cerebral abnormalities, candidates appear to be different for pure and complicated HSP subtypes. Most studies with larger cohorts of pure HSP found disease severity, expressed by the spastic paraplegia rating scale—SPRS (73), to be significantly correlated with diffusivity parameters at the anterior limb of internal capsule and corpus callosum, particularly the genu and body (42, 43). Such correlations are not found in complicated HSPs. In this last group, two studies with large SPG11 samples reported independently that the volume of certain cerebral regions correlates with disease-related disability (6, 38). Faber et al. indeed found that precentral/paracentral cortex thickness and deep nuclei volumes correlated not only with SPRS scores but also with disease duration in SPG11 (6). In line with that, the same authors showed that striatal DAT uptake also correlated inversely with disease severity and duration in SPG11 (63).

Spinal cord morphometry is another potential neuroimaging marker for HSPs. The first published studies failed to identify correlations between spinal cord area and disease severity (9, 42, 43, 70). However, this may be related to the small and rather heterogeneous HSP cohorts evaluated at the time. A recent study with a representative cohort of subjects with SPG11 revealed that cervical spinal cord area correlates inversely with SPRS scores and disease duration (6).

## Future Perspectives

There are few studies devoted to characterize neuroimaging abnormalities in HSP so far. Despite that, currently available data strongly support MRI as an important tool to assist in the exclusion of HSP-mimics, to guide genetic testing and to understand the pathophysiology of the disease. For the near future, much work still has to be done in order to fully appreciate how neuroimaging can help us to manage HSP. Considering the rarity of individual HSP subtypes, we should design multicentric studies to obtain representative and homogeneous cohorts of patients. It is rather probable that each specific HSP has its own structural signature (beyond CST damage), but this can only be clarified with properly designed studies.

HSP are very slowly progressive disorders, in such a way that clinical scales have poor sensitivity to track longitudinal changes. For such a rare disease, this greatly limits the design and execution of randomized clinical trials based solely upon clinical tools. So, the potential role of MRI as a state biomarker for HSP should be further explored. Nevertheless, we will require technical and quantitative harmonization between centers before attempting collaborative, multicenter natural history studies. This is certainly a challenging task, but recent efforts done for similar rare neurodegenerative disorders, such as ALS, are encouraging (74). Another relevant question is to identify the most promising neuroimaging markers. At this point, spinal cord morphometry, basal ganglia volumetry and corpus callosum diffusivity emerge as promising candidates, but they still need to be evaluated prospectively. Novel technical advances in spinal cord imaging now enable automated measurements of diffusivity parameters and separate GM vs. WM cross-sectional areas (75). For HSPs, these are other interesting candidates to be investigated.

## Author Contributions

FdG, TdR, JP, OB, MF, and LV conceived the research project. FdG, TdR, JP, OB, and MF organized the research project, reviewed, and critique the statistical analysis. FdG, TdR, MF, and LV executed the research project and wrote the first draft of the manuscript. FdG, TdR, and MF designed the statistical analysis and executed the statistical analysis. FdG, TdR, JP, OB, and MF. FdG, TdR, MF, and LV. JP, OB, and MF reviewed and critique the manuscript.

### Conflict of Interest Statement

The authors declare that the research was conducted in the absence of any commercial or financial relationships that could be construed as a potential conflict of interest.
